# Disentangling comorbidity in chronic pain: A study in primary health care settings from India

**DOI:** 10.1371/journal.pone.0242865

**Published:** 2020-11-30

**Authors:** Geetha Desai, Jaisoorya T. S., Sunil Kumar G., Manoj L., Gokul G. R., Aakash Bajaj, Thennarasu K., Santosh K. Chaturvedi

**Affiliations:** 1 Department of Psychiatry, National Institute of Mental Health and Neurosciences (NIMHANS), Bengaluru, India; 2 National Health Mission (Kerala), Thiruvananthapuram, India; 3 Department of Biostatistics, National Institute of Mental Health and Neurosciences (NIMHANS), Bengaluru, India; Institute of Economic Growth, INDIA

## Abstract

**Objectives:**

The study examined the prevalence, sociodemographic, and clinical correlates of chronic pain among primary care patients in the state of Kerala, India. It also examined the patterns and relationships of chronic physical and mental health conditions with chronic pain.

**Methods:**

This study is a cross-sectional survey conducted among 7165 adult patients selected randomly by a multi-stage stratified design from 71 primary health centers. The questionnaires administered included Chronic pain screening questionnaire, self-reported Chronic physical health condition checklist, Patient Health Questionnaire-SADS, The Alcohol Use Disorders Identification Test, Fagerström Test for Nicotine Dependence, WHO Disability Assessment Schedule and WHOQOL- BREF for Quality/Satisfaction with Life. The prevalence and comorbid patterns of chronic pain were determined. Logistic regression analysis and generalized linear mixed-effects model was employed to examine the relationship of chronic pain to socio-demographic variables and examined physical and mental health conditions.

**Results:**

A total of 1831 (27%) patients reported chronic pain. Among those with chronic pain, 28.3% reported no co-occurring chronic mental or physical illness, 35.3% reported one, and 36.3% reported multi-morbidity. In the multivariate analysis, patients with chronic pain when compared to those without had higher odds of being older, female, having lower education, not living with their family, greater disability, and poor satisfaction with life. Chronic pain was independently associated with both medical (hypertension, diabetes mellitus, tuberculosis, arthritis, and other medical illnesses) and mental health conditions (depressive disorders, anxiety disorders, and tobacco dependence). It showed a varying strength of association and additive effect with increasing number of co-occurring physical and mental illnesses.

**Conclusions:**

Chronic pain is a common condition among primary care attendees associated with significant burden of medical and mental health comorbidity. The findings highlight the need to incorporate treatment models that will ensure appropriate management to improve outcomes within the resource constraints.

## Introduction

Chronic pain is common and described as “pain lasting for more than 3–6 months or persisting beyond the usual course of an acute disease or after a reasonable time for healing to occur” [1]. It is associated with significant disability and reduced quality of life [1, 2]. In the largest study to date, which examined the cross-national prevalence of chronic pain conditions, 41.1% and 37.3% of subjects in developing and developed countries reported chronic pain [3]. The findings are similar to results from large surveys which have reported rates between 8–50% [3–9]. Studies examining chronic pain from India are however small, restricted to few institutions, or a specific sub-group (urban) with reported prevalence rates ranging from 9 to 29% [10–13]. Many socio-demographic factors like female gender, older age, lower socioeconomic status and cultural expressions of distress have been consistently reported to be associated with chronic pain [14–18]. The most common sites of pain reported are neck and back, with Indian studies also reporting the frequent occurrence of whole-body ache and pain in extremities. Many subjects also report pain in multiple sites, with site multiplicity being correlated with the severity of pain [19].

Elevated rates of physical and mental health comorbidity have been reported in patients with chronic pain, leading to poorer health outcomes and a greater burden [20–22]. Physical illnesses like arthritis, respiratory illness, cardiovascular disorders and diabetes mellitus are commonly associated with chronic pain [20, 21]. Similarly, psychiatric disorders like depression, anxiety and substance use disorders have been reported to be common comorbidities [23, 24]. The strength of relationship however vary, with some physical and mental health conditions being more strongly associated with chronic pain [20–22].

The association of co-morbidities with chronic pain needs to be studied further. Determining comorbid pattern in chronic pain while controlling for co-occurring physical and mental disorders is important as pain is now viewed both as an independent entity and also a symptom of underlying illness [25]. A study, which examined this in a nationally representative sample of adults in New Zealand, reported that only six out of fifteen examined physical/medical disorders were associated independently with chronic pain. Further, the presence of anxiety and or depression independently increased the odds of reporting chronic pain [21].

Majority of patients with chronic pain are treated in primary care, where multimorbidity is common [26, 27]. In developing countries, the proportion seeking services in primary care is likely to be higher as tertiary care pain clinics are non-existent. The higher prevalence of chronic pain in non-western communities implies that the burden of chronic pain in primary care in low- and middle-income countries (LAMIC) is likely to be higher. Management of these clinically challenging patients may be further compromised as in most developing countries, primary care physicians have a heavy patient load. In India, the estimated consultation time in primary care is 3 minutes [28]. Most primary health centers (PHC–(Government-owned general practices)), especially in rural and/or remote locations, are further constrained by minimum access to investigations. For medical professionals working in these resource constrained settings, an improved understanding of chronic pain and its independent comorbidities (physical and mental health) will help in focusing clinical care and improving outcomes. It is important as world over the management of chronic pain is generally unsatisfactory, with most patients reporting persistence of symptoms and disability for many years [22].

Since there is no available data from Indian settings, this study was done with the following objectives:

1. To report the prevalence, clinical characteristics and socio-demographic correlates of chronic pain among primary care attendees.
2. To examine the pattern of physical and mental comorbidity among subjects with chronic pain.
3. To examine whether each of the examined physical and mental disorders are independently associated with chronic pain.

This is a part of larger research study “Clinical presentations, mental health issues, disability and quality of life among primary care patients in the state of Kerala, India” funded by the National Health Mission (Kerala), a governmental organization which aims at decentralized health delivery in the community.

## Methodology

The study was approved by the Ethical committee of Government Medical College, Ernakulum (Approval No. 16/16, dated 26/2/2016). Subjects gave oral consent before being administered the questionnaire.

The study is a cross-sectional survey conducted in the year 2016 among patients attending government-owned primary health centers (PHCs) in the State of Kerala, India.

A representative sample of 7555 patients was obtained through multistage stratified random sampling method from 71 PHCs of the state. This sample was adequate to detect an expected prevalence of 30%, with a precision of 2.5%, with a confidence interval of 95% and a design effect of 2.5.

The block Public Relations Officers (Block PROs) of the National Health Mission (Kerala), who had postgraduate qualifications in social work administered the survey. They received training and were supervised by the medical officer in charge of the PHC.

The questionnaires were initially prepared in English, translated to Malayalam (the vernacular language), and back translated to ensure conceptual equivalence.

### Instruments

Socio-demographic profile (Age/sex/marital status/education/family structure/occupation/area of residence/socio-economic status) was assessed in the form of a checklist.

In addition, the following domains were explored:

### Chronic pain

Chronic pain (defined for purposes of this study as persistent pain for six months) was assessed using a questionnaire. If subjects reported that they had chronic pain, they were further asked about the duration, site, frequency, and interference with activities. The intensity of pain was classified as mild, moderate and severe based on interference with activities. Mild being no or infrequent interference, moderate being frequent interference in biological or occupational functioning and severe being significant interference in all activities.

### Chronic physical conditions

Chronic physical conditions for the purposes of the study was defined as any physical health condition that have lasted more than six months. Subjects were asked to report whether they had any chronic physical health conditions. The chronic physical conditions selected for exploration were chosen from findings of the POSEIDON study (prevalence of symptoms on a single Indian health care day on a national -wide scale). Chronic physical conditions which had an above 3% prevalence in the POSEIDON study with the exception of epilepsy (included) and anemia (excluded) were examined [29]. The authors by consensus excluded anemia as it was considered that our primary care patients may have difficulty to provide the necessary information. Epilepsy was included as it was part of the objectives of the larger study to examine common neuropsychiatric condition in primary care. The physical illnesses assessed included hypertension, diabetics mellitus, asthma, tuberculosis, chronic dermatological conditions, epilepsy and arthritis.

Assessment was by self-report. Although self-reporting may underestimate the prevalence of certain conditions, studies have reported good agreement with medical records [20]. Further, in India, medical records of treatment in PHCs are maintained by patients and not the general practitioner.

### Mental health conditions

The mental health conditions selected for inclusion were depressive disorders, anxiety disorders, alcohol and tobacco use. Anxiety, depressive disorders and somatoform are the commonest mental health conditions in primary care. Somatoform disorders have been excluded from assessment as it was deemed to have a significant overlap of its symptoms with pain conditions. Alcohol and tobacco are the commonest substances of abuse in India [30, 31].

The following assessment instruments were used:

- **Depression and Anxiety disorders-**The Patient Health Questionnaire-Somatic, Anxiety and Depressive Symptoms (PHQ-SADS) was used to assess depressive and anxiety disorders. The instrument is validated as a screening tool for assessing depression, anxiety, panic disorder, and somatization (common mental disorders) among primary care attendees [32]. Somatization was also assessed as a part of the larger study, but considering the significant overlap between chronic pain and somatization, it was not included for analysis [33]. Subjects in this study who screened positive for depression and other depressive disorders were categorized to have depressive disorders, and those with generalized anxiety disorder and panic disorder to have anxiety disorders.
- **Alcohol Use-** Alcohol use was assessed using Alcohol Use Disorders Identification Test (AUDIT). The screening instrument has 10-items, with hazardous use indicated by a score of 8 being used as a cut-off for this study [34].
- **Tobacco Use—**Fagerström Test for nicotine dependence was used to assess tobacco use. The instrument has been widely used for screening for nicotine dependence with excellent sensitivity, specificity and validity [35].

Subjects who had at least one mental health disorder on assessment (depressive disorders, anxiety disorders, hazardous alcohol use, or tobacco dependence) were categorized to have mental illness.

### Disability

The level of disability due to health conditions in the last one month was assessed using the 12 item World Health Organization Disability Assessment Schedule (WHODAS). Computed total scores are considered with higher scores indicating greater disability [36].

### Quality/satisfaction with life

Two items from World Health Organization Quality of Life Instrument brief version (WHOQOL-BREF) was used to screen for life satisfaction and quality of life [37].

### Statistical analysis

Statistical analysis was done using STATA (version 14). The data was expressed using descriptive statistics such as frequency and percentages for categorical variables and mean (SD) for continuous variables.

The prevalence, site, intensity, duration, and severity of chronic pain were determined. A full model of logistic regression analysis was done to determine the significant socio-demographic and outcome correlates (disability/quality of life/satisfaction of life) of chronic pain.

To examine the relationship of chronic pain to each of the examined physical and mental health conditions the following steps were employed:

1. The PHC weighted prevalence of chronic pain among subjects with each of the examined chronic physical illness and mental illness was calculated. The adjusted odds ratio (OR) of chronic pain in each chronic physical condition was calculated after controlling for other co-occurring physical disorders and socio-demographic variables. Similarly, the adjusted OR of chronic pain was determined for each of the examined mental health conditions after adjusting for other co-occurring mental illness and socio-demographic variables.
2. A step-wise generalized linear mixed-effects model (GLMM) was used to examine the independent relationship of chronic pain with examined chronic conditions:
  - In all examined physical conditions, an initial univariate analysis was done to report the relationship of each of the examined conditions with chronic pain.
  - In the second step, a binary logistic regression analysis for chronic pain was conducted for each of the physical conditions while controlling for socio-demographic variables.
  - In the third step, the relationship of chronic pain with a specific chronic physical condition, independent of the number of other co-occurring physical conditions was examined. It was done by measuring the overall count of the number of physical conditions suffered by each patient excluding the physical condition being examined. This measure was then used to ascertain whether the examined chronic physical condition remained significantly associated with chronic pain after removing the cumulative effect of number of co-occurring physical conditions.
  - A similar stepwise binary logistic regression analysis was done for all examined mental health conditions to examine which mental health condition remained significant after controlling for socio-demographic variables and total number of co-occurring mental health conditions.
  - To further disentangle the relationship of each of the examined chronic physical and mental health conditions to chronic pain, the analysis was controlled for both physical and mental illness load and further controlled for the interaction between physical and mental health conditions. The variation in the data due to sampling design (clustered at PHC level) was accounted for with the use of GLMM.

The risk associations were reported with Odds ratios (OR) with 95% confidence interval (CI).

## Results

Of the 7555 patients who were invited to participate, 390 (5.2%) did not provide consent to participate in the study. Among the 7165 patients who responded, 377 questionnaires had to be discarded as they had substantial missing responses, leaving 6788 (89.8%) questionnaires for analysis. Among them, 2344 (34.5%) were men with a mean age of 41.1 (SD-11.1) years.

Chronic pain was reported by 1831 (27%) patients [males-451 (19.2%); females-1380 (31.1%)]. The clinical characteristics of patients with chronic pain are described in **Table 1**. Majority of the patients had pain in the whole body and pain in the extremities. Most patients (67%) had pain for a duration between 6 months to 2 years, 51% of them experienced pain daily, and nearly 63% of the sample had moderate to severe intensity of pain.

**Table 1:** Clinical characteristics of subjects with chronic pain (N = 1831)*.

Clinical Characteristics	N (%)
**Site of Pain**	
Extremities	644 (34.1)
Whole body	410 (21.7)
Back	346 (18.3)
Head and Neck	236 (12.5)
Multiple sites	138 (7.3)
Abdomen	67 (3.5)
**Duration of pain**	
6–12 Months	711 (37.6)
1–2 Years	549 (29)
2–5 Years	236 (12.5)
5–10 Years	211 (11.2)
>10 Years	102 (5.4)
**Frequency of pain**	
Daily	963 (51)
Few days a week	489 (25.9)
Once in a week	183 (9.7)
Monthly	181 (9.6)
**Intensity of pain**	
Mild	619 (32.8)
Moderate	809 (42.8)
Severe	390 (20.6)

* Missing responses were excluded from analyses, so samples sizes do not add to 1831 for a few characteristics.

When patients with chronic pain were compared to those without pain on socio-demographic and other outcome variables using a full model of multivariate analysis, females, older individuals, those living away from family, having lower education, higher disability scores and poor satisfaction with life had higher odds of experiencing chronic pain **(Table 2)**.

**Table 2:** Socio-demographic & clinical correlates of subjects with chronic pain*.

	Chronic pain		
Correlates	Present N = 1831 n (%)	Absent N = 4957 n (%)	Odds Ratio (95% CI^‡^)
**Age** *(Mean ± SD)*	45.4±9.9	39.5±11.0	**0.972 (0.96–0.98)**
**Gender**			
Male	451 (19.2)	1893 (80.8)	**1.00**
Female	1380 (31.1)	3064 (68.9)	**1.95 (1.64–2.33)**
**Residence**			
Urban	769 (27.4)	2037 (72.6)	1.00
Rural	1086 (26.7)	2978 (73.3)	0.94 (0.82–1.07)
**Occupation**			
Unemployed	1123 (30.1)	2609 (69.9)	1.00
Employed	742 (23.7)	2394 (76.3)	1.03 (0.88–1.19)
**Education**			
<10 years	1543 (30.9)	3451 (69.1)	**1.00**
>10 years	308 (16.7)	1540 (83.8)	**0.79 (0.66–0.95)**
**Family Structure**			
Family	1656 (26.3)	4629 (73.7)	**1.00**
Alone	80 (38.3)	129 (61.7)	**1.60 (1.21–2.11)**
Institution/others	142 (32)	302 (68)	**1.63 (1.02–2.61)**
**Socioeconomic status**†			
Above Poverty Line	911 (25.4)	2672 (74.6)	1.00
Below Poverty Line	953 (28.8)	2354 (71.2)	0.985 (0.86–1.126)
**Marital status**			
Single	107 (14.3)	639 (85.7)	1.00
Married	1526 (27.0)	4125 (73.0)	0.98 (0.75–1.26)
Separated/Divorced	238 (43.5)	309 (56.5)	1.41 (0.97–2.079)
**Quality of Life**			
Good	886 (21.3)	3268 (78.6)	1.0
Average	606 (31.6)	1313 (68.4)	0.96 (0.74–1.24)
Poor	330 (50.5)	323 (49.5)	1.13 (0.96–1.34)
**Satisfaction with Life**			
Satisfied	798 (18.1)	3617 (81.9)	**1.0**
Average	495 (35.6)	897 (64.4)	**1.81 (1.53–2.17)**
Unsatisfied	528 (57.1)	397 (42.9)	**2.50 (2.09–3.12)**
**Disability Scores** *(Mean ± SD)*	7.7±8.6	2.9±6.5	**0.95 (0.94–0.96)**

* Missing responses were excluded from analyses, so samples sizes do not add to 6788 for a few characteristics.

† Socio-economic indicators of Government of India.

‡ Confidence interval.

Among subjects reporting chronic pain, 627 (16.8%) reported no co-occurring chronic physical illness, 770 (34.6%) reported one, while 434 (52.1%) reported more than two chronic physical conditions. Similarly, 1433 (77.4%) patients with chronic pain did not report any co-occurring mental illness, 264 (15.5%) reported one, and 134 (7.1%) reported more than one co-occurring mental illness. Collating both, among subjects with chronic pain, 28.3% reported no co-occurring chronic mental or physical illness, 35.3% reported one, and 36.3% reported more than one co-occurring physical or mental illness (multi-morbidity).

The proportion of patients reporting chronic pain in individual chronic medical illness varied from 31.0% among patients with chronic dermatological conditions to 69.6% for patients with arthritis. Correspondingly in the case of mental health conditions, the prevalence of chronic pain varied from 32.8% among patients with hazardous use of alcohol to 65.6% for those experiencing depression. Further, the risk ratios of occurrence of chronic pain in each physical and psychiatric disorder after adjusting for socio-demographic variables and co-occurring physical and mental health conditions showed arthritis and depressive disorders to have the highest risk of reporting chronic pain among examined physical and mental health disorders. In contrast, epilepsy, chronic dermatological conditions, and hazardous use of alcohol were not associated with chronic pain **(Table 3)**.

**Table 3:** Prevalence of chronic pain in physical and mental disorders with risk ratios adjusted for other physical/mental illness and socio-demographic variables.

**Physical Illness (N)**	**Prevalence of Chronic Pain n (%)**	**Adjusted for Other co-occurring Physical Illness OR (95%CI)**	**Adjusted for Other co-occurring Physical Illness and socio-demographic variables OR (95%CI)**
Hypertension (1280)	582 (45.4)	**2.34 (2.025, 2.702)**	**1.69 (1.441, 1.983)**
Diabetes Mellitus (1137)	493 (43.4)	**1.85 (1.587, 2.147)**	**1.45 (1.229, 1.713)**
Epilepsy (40)	18 (45)	1.49 (0.753, 2.936)	1.27 (0.593, 2.733)
Tuberculosis (46)	25 (54.3)	**2.96 (1.580, 5.540)**	**3.6 (1.794, 7.234)**
Dermatology conditions (184)	57 (31.0)	**1.43 (1.001, 2.039)**	1.33 (0.898, 1.96)
Arthritis (191)	133 (69.6)	**9.35 (6.596, 13.245)**	**7.09 (4.845, 10.368)**
Asthma (158)	69 (43.7)	**2.26 (1.592, 3.201)**	**1.74 (1.185, 2.545)**
Others (1158)	395 (35.0)	**1.95 (1.641, 2.314)**	**1.82 (1.511, 2.188)**
**Mental Illness (N)**	**Prevalence of Chronic Pain n (%)**	**Adjusted for Other Mental Illness OR (95%CI)**	**Adjusted for Other mental Illness and Socio-demographic variables OR (95%CI)**
Alcohol hazardous use (119)	39 (32.8)	0.95 (0.619, 1.452)	1.06 (0.672, 1.688)
Tobacco dependence (273)	102 (37.4)	**1.47 (1.101, 1.949)**	**1.98 (1.438, 2.725)**
Depression (350)	230 (65.6)	**3.98 (3.075, 5.156)**	**3.36 (2.542, 4.441)**
Anxiety (226)	147 (65.0)	**2.61 (1.894, 3.592)**	**2.17 (1.526, 3.086)**

The prevalence of chronic pain increased with an increasing number of physical and mental health conditions. In patients with no co-occurring physical illness the prevalence of chronic pain was 16.8%, with one physical illness the prevalence was 34.7% and with more than one physical illness it was 52.1%. Similarly, in patients with no co-occurring mental health conditions the prevalence of chronic pain was 23.8%, with one the prevalence was 49.7% and with more than one it was 60.6%.

**Table 4** depicts the relationship of chronic pain when examined with physical and mental health illness using generalized linear mixed-effects model. Chronic physical conditions significantly associated with chronic pain in the unadjusted analysis were hypertension, diabetes mellitus, tuberculosis, arthritis, asthma, and other medical illnesses. Similarly, mental health conditions associated with chronic pain were depressive disorders, anxiety disorders, and tobacco dependence. This association persisted even after a stepwise analysis to control for possible confounding variables in the following domains: socio-demographic variables, the total number of physical illnesses; the total number of mental illness; the combination of total number of physical and mental health conditions; and interaction of physical and mental health conditions. The only exception was asthma, which no longer showed association when controlled for socio-demographic variables and the total number of mental health conditions but was significant in other models.

**Table 4:** Generalized linear effects models of physical and mental health comorbidity in patients with chronic pain.

Disease	Unadjusted (U)		U +Socio Demographic variable (SD)		U+SD+ Total number of Physical illness (PT)		U+SD+ Total number of Mental illness (MT)		U+SD+PT+MT		U+SD+PT+MT+ Interaction (I)	
	OR (95%CI)	p value	OR (95%CI)	p value	OR (95%CI)	p value	OR (95%CI)	p value	OR (95%CI)	p value	OR (95%CI)	p value
Hypertension	2.61 (2.279,2.984)	<0.001	1.66 (1.424,1.935)	<0.001	1.59 (1.364,1.861)	<0.001	1.64 (1.402,1.913)	<0.001	1.58 (1.35,1.85)	<0.001	1.58 (1.353,1.854)	<0.001
Diabetes Mellitus	2.21 (1.921,2.544)	<0.001	1.46 (1.245,1.713)	<0.001	1.39 (1.182,1.636)	<0.001	1.47 (1.249,1.726)	<0.001	1.41 (1.196,1.661)	<0.001	1.41 (1.198,1.663)	<0.001
Epilepsy	1.29 (0.657,2.517)	0.463	1.09 (0.509,2.337)	0.823	1.21 (0.564,2.603)	0.624	0.96 (0.447,2.075)	0.923	1.08 (0.499,2.32)	0.852	1.01 (0.466,2.2)	0.976
Tuberculosis	2.8 (1.52,5.154)	0.001	3.29 (1.649,6.584)	0.001	3.49 (1.737,6.997)	<0.001	2.87 (1.431,5.748)	0.003	3.02 (1.506,6.041)	0.002	2.81 (1.393,5.654)	0.004
Dermatological conditions	1.14 (0.809,1.593)	0.462	1.15 (0.785,1.678)	0.476	1.31 (0.896,1.928)	0.162	1.15 (0.781,1.683)	0.486	1.3 (0.88,1.913)	0.188	1.3 (0.879,1.916)	0.19
Arthritis	7.99 (5.691,11.222)	<0.001	5.86 (4.029,8.512)	<0.001	6.99 (4.781,10.22)	<0.001	5.65 (3.857,8.266)	<0.001	6.69 (4.541,9.842)	<0.001	6.62 (4.491,9.76)	<0.001
Asthma	2 (1.427,2.79)	<0.001	1.47 (1.008,2.131)	0.045	1.73 (1.181,2.523)	0.005	1.34 (0.915,1.958)	0.133	1.57 (1.065,2.315)	0.023	1.58 (1.071,2.321)	0.021
Others	1.55 (1.311,1.822)	<0.001	1.53 (1.277,1.83)	<0.001	1.75 (1.461,2.107)	<0.001	1.48 (1.236,1.78)	<0.001	1.7 (1.412,2.047)	<0.001	1.69 (1.401,2.03)	<0.001
Alcohol hazardous use	1.06 (0.706,1.58)	0.791	1.3 (0.836,2.018)	0.245	1.31 (0.84,2.041)	0.234	0.97 (0.614,1.526)	0.889	1 (0.634,1.58)	0.998	0.98 (0.621,1.546)	0.93
Tobacco Dependence	1.43 (1.09,1.875)	0.01	2.04 (1.499,2.774)	<0.001	2.2 (1.615,3.005)	<0.001	1.74 (1.27,2.387)	0.001	1.9 (1.38,2.605)	<0.001	1.85 (1.343,2.542)	<0.001
Depression	5.12 (3.997,6.558)	<0.001	4.07 (3.112,5.324)	<0.001	3.62 (2.76,4.748)	<0.001	3.53 (2.684,4.64)	<0.001	3.12 (2.367,4.115)	<0.001	3.12 (2.37,4.118)	<0.001
Anxiety	4.24 (3.144,5.726)	<0.001	3.28 (2.346,4.573)	<0.001	3.08 (2.198,4.31)	<0.001	2.41 (1.705,3.396)	<0.001	2.28 (1.613,3.23)	<0.001	2.32 (1.64,3.274)	<0.001

## Discussion

The prevalence of chronic pain among our primary care attendees was 27%. Studies examining chronic pain have reported a wide variation in prevalence (8–50%). Methodological aspects including the pain condition examined, settings (general population/primary care/hospitals), methods of assessment (self-report/screening/structured interview) and socio-cultural differences have been postulated to explain this variation. Despite this, our prevalence rate is broadly comparable to the rates reported from large studies among primary care patients. Data from the WHO Collaborative study of Psychological Problems in General Health Centre has reported the prevalence of chronic pain across 15 centers in 14 countries (including India) to be 22% (range 5–33%) [4]. A systematic review on prevalence of chronic pain in developing countries including studies in general population reported a rate of 18% [38]. Reporting of higher rates are also not uncommon with 42% of German primary care attendees reporting chronic pain [39]. Existing studies from India among smaller samples which examined patients from a single primary health centre [12] or in urban population [11, 13] have reported rates of 13–29%. Studies done in India have reported whole body ache and pain in extremities as the most common sites of pain [10–12], which is similar to our study findings.

Our study replicated the consistent association of chronic pain with older age [14–16, 40]. Subjects who are older are more likely to have physical and mental comorbidities, all of which were independently associated with chronic pain. Increasing age is also associated with a higher likelihood of having experienced an injury [14]. Female subjects in our study had a higher prevalence of chronic pain, as reported prior [14–16, 41–44]. Both biological and psychosocial factors have been reported to contribute to the greater reporting of chronic pain among women. While the mechanisms remain to be clarified, various studies have reported lower-pain thresholds, maladaptive coping, with some evidence of the role of estrogen and genetics, to explain this heightened risk [14, 41–44]. Our study did not find any relationship of chronic pain with socioeconomic status. However, our subjects with lower education had a higher risk of reporting chronic pain, with previous studies suggesting that chronic pain was correlated with lower education, income inequalities, and neighborhood deprivation [14, 45]. Our patients who were living alone were more likely to report chronic pain. Robust links have been reported between loneliness, pain, and depression [46]. Our subjects with chronic pain had poor life satisfaction and higher disability scores as has been reported in previous studies [15, 17].

Our subjects with chronic pain, had high rates of comorbidity with chronic physical illnesses and mental health conditions. High rates of comorbidity have been consistently reported in previous studies and has been associated with poorer outcomes and greater disability [20, 21, 47–49]. More concerning was the finding that approximately one in three of our patients with chronic pain had multimorbidity. Multimorbidity is associated with increased health care utilization, complex pharmacological regimes, higher costs, poorer recovery and higher mortality [50].

To disentangle the effect of various co-occurring conditions on chronic pain we have taken two approaches. The first, we assumed that the pathophysiological effects of a specific disorder on chronic pain can be estimated after controlling the differential effects of other coexisting disorders. In the second, we assumed the effects of pathophysiology of a single disease on chronic pain is better discerned after adjusting cumulative effect of number of disorder and their interactions. Both these approaches have been attempted in the past and has its votaries and disadvantages [51]. Given this, we have attempted to disentangle whether each of the examined conditions have independent effects using both methods.

In doing so, among the most commonly encountered physical illness among our primary care attendees, we report that hypertension, diabetes mellitus, tuberculosis, arthritis, asthma and others (residual category of varying chronic physical conditions grouped together) to have independent effects. Similarly, when mental health conditions were examined, depression, anxiety and tobacco use had independent association. Our findings are robust as we could replicate it at all stages of our stepwise approach to control for socio-demographic factors, physical/mental illness load and interaction between physical and mental illness. Further, the results held true when we controlled for occurrence of individually co-occurring physical/mental health conditions. The increased expression of chronic pain with an increased number of physical disorders and mental health conditions suggests an additive effect. The strength of association of pain with the examined individual disorders varied in each step of the logistic regression analysis possibly implying that the expression of chronic pain is influenced by biological, psychological, and social factors that may have both independent, additive, and interaction effects.

Subjects with arthritis had the highest odds of reporting chronic pain among our primary care patients. Chronic pain is common among subjects with arthritis being closely linked to disability, functional recovery and quality of life [52]. Both central and peripheral pain mechanisms have been reported to mediate expression of pain in patients with arthritis [53]. The independent association of diabetes mellitus and hypertension with chronic pain among our patients have been reported prior [54, 55]. Shared risk factors of obesity, low levels of physical activity, poor muscle mass and low-grade systemic inflammation help explain this association. In addition in hypertension, it has been hypothesized that mechanisms impaired in chronic pain dysregulate both pain responsiveness and blood pressure [56]. Tuberculosis, common among our primary care patients, was associated with chronic pain. In addition to chronic pain being the presenting symptom in musculoskeletal manifestations of tuberculosis, pain can also be the side-effect of certain anti-tubercular drugs [57, 58]. Another disorder which had independent association with chronic pain in most of our examined models with chronic pain was asthma. This finding has been reported prior, with breathlessness and consequent overuse of muscles involved in breathing heightening the experience of pain [59]. In variance to prior studies, our patients with chronic pain did not report an association with chronic dermatological conditions and epilepsy [60, 61]. The association of chronic pain have been reported with specific dermatological conditions like psoriasis, suggesting that our assessment by grouping all chronic dermatological conditions as a single entity may have not have appropriate to examine this relationship [60]. We speculate that patients attending primary care and self-reporting epilepsy may have been regularly on anti-epileptic medications which are by themself useful in chronic pain thus reducing its likelihood. Studies reporting the association of epilepsy with chronic pain have reported physical inactivity, bone injuries and high co-occurrence of migraine to mediate the relationship between epilepsy and chronic pain [61].

Among our subjects with chronic pain, depression and anxiety disorders had independent effects after controlling for confounding variables, including co-occurring physical and mental illness. The strength of the association was stronger for depression when compared to anxiety. In non-psychiatric clinical settings, including primary care, the predominant presentation is often an admixture of both anxiety and depressive symptoms. A strict syndromic separation may not be possible or appropriate. Hence our strategy of simultaneously examining both disorders and statistically controlling the occurrence of each other has improved the robustness of the finding of both its independent effects and relative strength of association. Presence of chronic pain with depressive and anxiety disorders has been commonly reported before to poorer outcomes and greater disability [4, 49, 62–64]. The significant association of our subjects with chronic pain to tobacco dependence replicates similar findings [65, 66]. A number of mechanisms, including self-medication, have been explained to understand the association of chronic pain and tobacco use [66]. Among our subjects, alcohol hazardous use was not associated with chronic pain. This contradicts previous studies which have reported a relationship [67–70]. Our sample was predominantly female and in India, alcohol use among females use is less than 5% [71]. This possibly explains the limited number of hazardous users among our subjects and the lack of association with alcohol use in our study. Most studies to date have examined the relationship one or a few major mental illnesses with chronic pain and have reported association [3, 23, 43]. The findings of common co-occurrence, multimorbidity and independent effects need to be highlighted as the evaluation and detection of psychiatric comorbidity in primary care remains limited, with the non-detection rate of up to 60% [72].

The study findings have important public health implications for India. The global burden of diseases has reported pain, and pain-related disorders are among the leading causes of current and future disability globally. The direct and indirect costs of pain have not been estimated in India, but annual costs estimated in the USA are huge at $560–$635 billion [73–75]. Given that the prevalence estimates and correlates of chronic pain reported in this study are consistent with those noted in other studies mostly from high-income countries, the outcome variables, both individual and social, at least on some levels may have similar costs. If so, the Indian public health system is ill-equipped to address this issue. The shortfall of required human resources means that the consultations time is brief [28]. This will invariably create gaps in recognizing and diagnosing comorbidities, both physical and mental, which will impede progress in pain management. Health administrators in India need to consider development of stepped care and collaborative care models for the management of chronic pain in primary care. Training nurses and other non-medical health care professionals in primary care for initial comprehensive assessment may help. Greater awareness and improving competency among primary care physicians of comorbid patterns especially mental health issues is required. The establishment of pain clinics in tertiary health care institutions need to be considered as a priority.

The study had its limitations. The study was cross-sectional; hence causality has not been inferred. Only a limited number of medical illnesses, which were deemed by consensus to be commonly occurring in our general practices, were examined. Important covariates like treatment variables were not examined. Only limited questions were used to assess life-satisfaction. Assessment of mental illness was using validated screening instruments. We acknowledge that screening questionnaires are not sufficient for diagnosis but identifies the major symptoms with the cut-off score approximating the diagnosis. However, we examined a large sample from multiple institutions using structured instrument validated in primary care. In addition to examining multiple physical and mental illness simultaneously, we statistically controlled for co-occurrence of other physical and mental illness, making it possible to report the independent relationship of various commonly occurring comorbidities with pain robustly.

## Conclusions

Chronic pain is common among primary care patients in Kerala, India. It is independently associated with a range of medical and mental health conditions. The strength of association varied, with a higher disability and additive effect with increasing number of physical and mental co-morbidities. Addressing this is thus a priority, with policy level strategies which takes into account the resource constrained primary health care settings. Stepped care and collaborative care approaches involving nurses and non-medical health care professionals need to be considered. The is also a need to improve awareness and competency of primary health care doctors in managing pain as an independent disorder, or as symptom of underlying illness, with its associated physical and mental health comorbidity.

## Supporting information

S1 Data (XLSX)
